# Over-Insurance

**Published:** 1914-07-18

**Authors:** 


### OVER-INSURANCE.

One of the really serious results of National Insurance has been the staggering blow which its inauguration has dealt to the Friendly Societies. To placate the latter, the Chancellor of the Exchequer was fertile in assurances and in safeguards, though shrewd judges prophesied even in 1910 that the Act meant their ruin. One of the expedients to retain the support of these important, because vote-swaying, corporations was a clause enabling the National Insurance members to join also, at their option, for extra benefits, paying the premiums direct to the societies. This proviso, it was expected, would bring to the Friendly Societies great accessions of membership and prosperity. In actual fact, the very reverse has been the case. The upshot of the permission has been that large numbers of the insured persons in approved societies connected with the old Friendly Societies are now "over-insured"—that is, insured so heavily that they actually receive as much, or more, money when unable to work as they do in health. "Over-insurance" is a condition which should be forbidden by law in connection with national health, just as it is by law in connection with fire insurance or insurance of shipping. It is disastrous to the State by demoralising its citizens, and the aim of all prudent statesmanship should be to discourage it.

Human nature being much the same in all classes, and in all races for that matter, the inevitable result is that malingering is directly encouraged, and the calls upon the funds are therefore much greater than the actuarial estimates. Mere malingering, however, can be detected and checked by an adequate medical service, and does not constitute the chief drain that is unstopped by over-insurance. Even more important is the removal of the main incentive to recovery, the discouragement of pluck and energy, the inducement to laziness and neurasthenic conditions. "Why should I hurry to get well," says the insured to himself, "if it means getting less money and having to work instead of doing nothing?" Over-insurance, permitted, and even encouraged, under the Insurance Act, is making even greater mischief than rendering several Friendly Societies more or less insolvent. It is sapping the national character at the roots, destroying insidiously but rapidly the virility, the energy, the honesty of the insured classes. The laws rightly prohibit the issuing of life insurance policies to any except those who can show an "insurable interest" in the life proposed. So, too, they ought to prevent, instead of expressly encouraging, the evil of over-insurance. This is no imaginary or purely theoretical evil: it was foretold with confidence long ago, during the discussions on the National Insurance Act, and it is now a substantiated fact from which there is no escape, and a very serious one at that.

It has been stated that several Friendly Societies are more or less insolvent; a statement with which no one behind the scenes is likely to quarrel, but one whereof we happen to be able to supply an apt proof. A circular has been brought to our notice, issued to its members by a very old and famous Friendly Society. In this circular it is intimated that, "in consequence of the extraordinarily heavy claims upon the Sickness Fund of the Society made by members during the year 1913 . . . the General Committee have been compelled *for the first time in the long history of the Society* to . . . declare a levy upon the members towards making good the deficiency." Following this is an intimation of the amount of the graduated levy, *equal to a third of the ordinary quarterly subscription,* which the particular member to whom the circular is addressed will have to pay. If this is what has happened to a Friendly Society as a result of National Insurance, the prospects of approved societies under the Act are black indeed; for the same causes are of course at work there, in an accentuated form, and must inevitably produce results, similar in kind but probably even greater in degree. Those who have read our National Insurance articles know that this is indeed the case, and that a fair proportion of these approved societies are within measurable distance of bankruptcy. The Act provides for reduced benefits in such cases, but the process will not be popular.

This disastrous state of affairs speaks for itself. To some extent it may be due to the admission of inferior lives into the Approved Society started by this Friendly Society, and to the taking up by these new members of the extra Friendly Society benefits. But we cannot believe that this explains the whole, or even the major part, of the unusual drain upon the funds. Undoubtedly the chief share is due to the Insurance Act, or rather to the over-insurance which it permits. From the grudging admissions in the House of Commons of the Chancellor it has been ascertained that many of the approved societies are insolvent, or barely solvent, though some of them have margins on the right side of their balance-sheets. Could any more eloquent comment be made upon the folly of ill-constructed and ill-considered legislation than the pass to which the Insurance Act has reduced, not only its own mechanism, but the splendid Friendly Societies which have done so much in the past towards encouraging thrift, self-help, and security in illness for the best types of the industrial population?

